# Prevalence, Comorbidities, and Mortality of Obesity Classes 4 and 5 in Adults

**DOI:** 10.1002/osp4.70171

**Published:** 2026-07-22

**Authors:** Katelynn Tran, Xinlian Zhang, Phillipp Hartmann

**Affiliations:** ^1^ Department of Pediatrics University of California San Diego La Jolla California USA; ^2^ Division of Biostatistics and Bioinformatics Herbert Wertheim School of Public Health and Human Longevity Science University of California San Diego La Jolla California USA; ^3^ Division of Gastroenterology Hepatology & Nutrition Rady Children's Hospital San Diego San Diego California USA

**Keywords:** adulthood obesity, cardiovascular risk factors, insulin resistance, MASLD, metabolic syndrome

## Abstract

**Introduction:**

Adult obesity has reached epidemic proportions globally. Although the prevalence and comorbidities of standard obesity classes (classes 1–3, BMI 30–49.9 kg/m^2^) have been described, the characteristics of obesity classes 4 and 5 (BMI ≥ 50 to < 60 kg/m^2^ and BMI ≥ 60 kg/m^2^, respectively), representing the most extreme end of the obesity spectrum, remain poorly defined.

**Objective:**

The objective of this study was to determine the prevalence of obesity classes 4 and 5, or extremely severe obesity, in adults over time and to investigate the relationship between extremely severe obesity and metabolic comorbidities and mortality.

**Methods:**

Data from nationally representative samples from National Health and Nutrition Examination Surveys from 2000 through 2023 involving 54,793 adults aged 18–65 years in the US were analyzed, including mortality data from 43,454 adults.

**Results:**

The prevalence of obesity classes 4 and 5 increased by over 250% from 0.6% in 2000 to 2.2% in 2023 (*p* < 0.001). Extremely severe obesity was particularly prevalent among females and non‐Hispanic Blacks. Subjects with obesity classes 4 and 5 had an odds ratio of 6.7–78.7 and 2.1–10.9 of metabolic comorbidities, and a 10‐year mortality hazard ratio of 2.2 (95% CI 1.6–3.0) and 1.8 (95% CI 1.3–2.4) compared with subjects without obesity and with obesity classes 1–3, respectively (all *p* < 0.001).

**Conclusions:**

The findings highlight the rise of a so far insufficiently characterized obesity entity, extremely severe obesity, and underline the strong association of extremely severe obesity with severe metabolic complications including death. These results emphasize the need for immediate public health interventions to address obesity, particularly obesity classes 4 and 5.

## Graphical abstract

Obesity classes 4 and 5 are associated with higher mortality than lower obesity classes or no obesity.

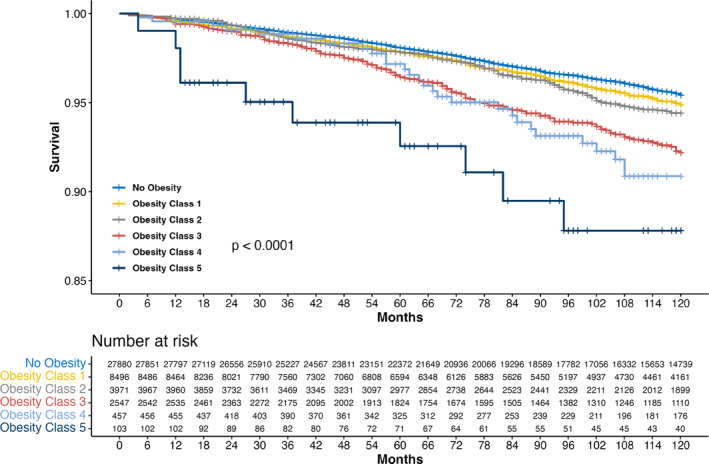

**Abbreviations**
BMI: Body Mass Index
CAP: Controlled Attenuation Parameter
CDC: Centers for Disease Control and Prevention
CI: confidence interval
CRP: C‐reactive Protein
GLP‐1RA: Glucagon‐like Peptide‐1 Receptor Agonist
HbA1C: Hemoglobin A1C
HDL: High‐Density Lipoprotein
HOMA‐IR: Homeostatic Model Assessment of Insulin Resistance
HR: Hazards Ratio
LDL: Low‐Density Lipoprotein
MASH: Metabolic Dysfunction‐Associated Steatohepatitis
MASLD: Metabolic Dysfunction‐Associated Steatotic Liver Disease
NHANES: National Health and Nutrition Examination Survey
T2DM: Type 2 Diabetes Mellitus

## 1 Introduction

The World Health Organization states that adult obesity worldwide has more than doubled since 1990; as of 2022, at least 2.5 billion adults are overweight with 890 million living with obesity [1]. Currently, 40.3% of adults in the US have obesity and this number is expected to rise to 50% by 2030 [2, 3, 4]. This exponential rise in obesity is considered a national health crisis as numerous studies in the last decades have shown that obesity is linked to comorbidities such as type 2 diabetes mellitus (T2DM), metabolic dysfunction–associated steatotic liver disease (MASLD), and cardiometabolic syndromes that increase healthcare costs and mortality and reduce quality of life [5, 6, 7]. The rising obesity rates in the setting of a national shortage in treatments such as Glucagon‐like peptide‐1 receptor agonists (GLP‐1RAs) compound the crisis [8].

Individuals with extremely severe obesity represent a distinct and particularly high‐risk population in the metabolic and bariatric surgery literature [9, 10]. Perioperative morbidity and mortality following bariatric procedures are substantially elevated in patients with BMI ≥ 50 kg/m^2^ compared with those with lower obesity classes, including higher rates of anastomotic leaks, pulmonary embolisms, prolonged ventilator dependence, and intensive care unit admission [9, 10]. This finding is substantiated by a greater obesity‐associated disease burden and more challenging surgical anatomy with extreme forms of obesity [11]. Weight loss outcomes after metabolic and bariatric surgery, while meaningful, are also more limited in this population, with lower rates of achieving a BMI below 40 kg/m^2^ compared with patients with class 1–3 obesity [12]. Despite these challenges, metabolic and bariatric surgery remains the most effective and durable treatment for extremely severe obesity, and guidelines from the American Society for Metabolic and Bariatric Surgery support the use of metabolic and bariatric surgery in this population [11].

Most recently, an extension to the traditional obesity classes 1–3 in children has been proposed with obesity class 4 defined as BMI ≥ 160% to < 180% of the 95th percentile for sex and age, and class 5 obesity defined as BMI ≥ 180% of the 95th percentile for sex and age [13]. Pediatric obesity classes 4 and 5 were associated with a significantly higher prevalence of MASLD and higher fibrosis stages, prediabetes/diabetes, severe insulin resistance, and other cardiometabolic risk factors including metabolic syndrome, low high‐density lipoprotein cholesterol and increased blood pressure compared with milder forms of obesity [13]. Although the prevalence and comorbidities as well as mortality of obesity classes 1–3 have been investigated in adults [2, 3, 4, 14], these characteristics have not been explored in adult obesity classes 4 and 5 (or extremely severe obesity).

The hypotheses were that extremely severe obesity in adults has increased in prevalence over the past two decades and that cardiometabolic complications and mortality would be higher in obesity classes 4 and 5 compared with less severe forms of obesity and no obesity.

## 2 Methods

### 2.1 Data Source

The National Health and Nutrition Examination Survey (NHANES) is a nationally representative, prospective survey conducted on U.S. residents every 2 years. Managed by the National Center for Health Statistics and the Centers for Disease Control and Prevention (CDC), NHANES aims to assess the health and nutritional status of the US population. As part of the survey, participants provide demographic information, undergo a physical examination, undergo blood and imaging studies, and complete validated questionnaires about health behaviors [15]. For the present analysis, the NHANES datasets 1999–2000 (referred to as the “NHANES 2000” dataset), 2001–2002 (“NHANES 2002”), 2003–2004 (“NHANES 2004”), 2005–2006 (“NHANES 2006”), 2007–2008 (“NHANES 2008”), 2009–2010 (“NHANES 2010”), 2011–2012 (“NHANES 2012”), 2013–2014 (“NHANES 2014”), 2015–2016 (“NHANES 2016”), 2017–2018 (“NHANES 2018”), 2017–2020 (“NHANES 2020”), and 2021–2023 (“NHANES 2023”) were obtained using the “nhanesA” library in R [16]. NHANES mortality data from 2000 to 2018 were downloaded from https://www.cdc.gov/nchs/data‐linkage/mortality‐public.htm.

### 2.2 Definition of Different Classes of Obesity

The various obesity classes were defined as follows: class 1 BMI ≥ 30 kg/m^2^ to < 35 kg/m^2^, class 2 BMI ≥ 35 kg/m^2^ to < 40 kg/m^2^, class 3 BMI ≥ 40 kg/m^2^ to < 50 kg/m^2^, class 4 BMI ≥ 50 kg/m^2^ to < 60 kg/m^2^, and class 5 BMI ≥ 60 kg/m^2^ [17].

### 2.3 Definitions of Conditions of Metabolic Dysfunction

Metabolic dysfunction‐associated steatotic liver disease (MASLD) was defined as per Rinella et al. [18]. After ruling out other liver conditions including hepatitis B and C, autoimmune hepatitis and other liver diseases, based on questionnaires and serology as available, MASLD was defined by presence of hepatic steatosis as well as at least one of five cardiometabolic criteria: BMI ≥ 25 kg/m^2^ (BMI ≥ 23 kg/m^2^ for Asians) or waist circumference > 94 cm for males and > 80 cm for females; prediabetes/diabetes per hemoglobin A1c ≥ 5.7%, fasting serum glucose ≥ 100 mg/dL, or prior diagnosis of prediabetes/diabetes; arterial hypertension per blood pressure ≥ 130/85 mmHg or on antihypertensive treatment; hypertriglyceridemia per fasting triglyceride ≥ 150 mg/dL or on lipid‐lowering treatment; or low HDL of ≤ 40 mg/dL for males or ≤ 50 mg/dL for females [18]. Hepatic steatosis was defined by transient elastography using a cutoff of 302 dB/m per controlled attenuation parameter (CAP) [19]. Hepatic steatosis grades 1+, 2+, and 3 were defined by a CAP of ≥ 302 dB/m, ≥ 331 dB/m, and ≥ 337 dB/m, respectively, as described [19]. Hepatic fibrosis stages 2 +, 3 +, and 4 were defined by a cutoff of 8.2 kPa, 9.7 kPa, and 13.6 kPa, respectively, as described [19].

Metabolic syndrome was diagnosed according to de Alberti et al. [20] if at least three out of five cardiometabolic criteria were met: waist circumference > 94 cm for males and > 80 cm for females; prediabetes/diabetes per hemoglobin A1c ≥ 5.7%, fasting serum glucose ≥ 100 mg/dL, or prior diagnosis of prediabetes/diabetes; arterial hypertension per blood pressure ≥ 130/85 mmHg or on antihypertensive treatment; hypertriglyceridemia per fasting triglyceride ≥ 150 mg/dL or on lipid‐lowering treatment; or low HDL of ≤ 40 mg/dL for males or ≤ 50 mg/dL for females [20].

For the purposes of this study, the prevalence of prediabetes was determined by hemoglobin A1c ≥ 5.7% and < 6.5% and prior history of prediabetes; the prevalence of diabetes mellitus type 2 was determined by hemoglobin A1c ≥ 6.5% and prior history of diabetes. Homeostatic model assessment for insulin resistance (HOMA‐IR) is the product of fasting insulin [μU/mL] * fasting glucose [mg/dL])/405 [21]. Insulin resistance was defined as HOMA‐IR ≥ 2.5 [21]. Severe insulin resistance was defined by fasting insulin levels ≥ 50 μU/mL [22].

### 2.4 Statistical Analysis

Results are presented as medians with first and third quartiles unless specified otherwise. Comparisons between the two groups for continuous variables were made using the Wilcoxon–Whitney–Mann rank‐sum test. For comparisons among three or more groups, the Kruskal–Wallis test was used. If the Kruskal–Wallis test indicated statistical significance, pairwise Wilcoxon–Whitney–Mann rank‐sum tests with Holm correction were conducted for three to five groups and False Discovery Rate (FDR) correction for six or more groups, and the significant adjusted *p* values were reported in the figures or tables.

Comparisons between two or more groups for prevalence data were made using the Pearson”s Chi‐squared test, and for more than two groups pairwise comparisons with Holm or FDR corrections were performed as above. All statistical tests were two‐sided. Pearson correlation analyses were conducted to examine relationships between variables, with *p* values adjusted for multiplicity using Holm/FDR correction where indicated. Multiple logistic regression and odds ratio calculations were carried out using the “stats” and “questionr” libraries in R, respectively. Kaplan‐Meier curves, hazard ratio, and additional mortality calculations were prepared using the “survival” library in R. A *p* value of less than 0.05 was considered statistically significant for all tests. Statistical analyses were performed using R statistical software, R version 2023.09.1 for Mac, the R Foundation for Statistical Computing.

## 3 Results

### 3.1 Demographics and Clinical Data

Table 1 summarizes the demographic, physical examination, laboratory, and imaging data of the entire cohort and mortality cohort from the 2000 to 2023 NHANES dataset. The entire cohort consists of 54,793 adults aged 18–65 years with 43,454 being a subset for whom survival data were available. Both cohorts represented males (47.7%; 47.9%) and females (52.3%; 52.1%) equally. The median age was 40 years, with both cohorts representing a racially diverse population including Mexican Americans (18.2%; 20%), other Hispanics (9.1%; 8.6%), non‐Hispanic Whites (38.6%; 38.6%), non‐Hispanic Blacks (22.6%; 22.7%), and other races including multiracial backgrounds (11.4%; 10.1%). The median BMI was 27.8 kg/m^2^ and 27.6 kg/m^2^.

**TABLE 1:** Demographic, physical exam, laboratory, and imaging data of the entire 2000–2023 study population as well as the mortality cohort.

	*n*	Entire cohort 2000–2023, *n* = 54,793	*n*	Mortality cohort, *n* = 43,454
Gender:	54,793		43,454	
Male		26,113 (47.7%)		20,835 (47.9%)
Female		28,680 (52.3%)		22,619 (52.1%)
Age [years]	54,793	40.0 [28.0; 53.0]	43,454	40.0 [27.0; 52.0]
Ethnicity/Race:	54,793		43,454	
Mexican American		9995 (18.2%)		8686 (20.0%)
Other Hispanic		5000 (9.1%)		3735 (8.6%)
Non‐Hispanic White		21,164 (38.6%)		16,759 (38.6%)
Non‐Hispanic Black		12,370 (22.6%)		9876 (22.7%)
Other race—Incl. multi‐racial		6264 (11.4%)		4398 (10.1%)
Height [cm]	54,793	167.0 [160.0; 175.0]	43,454	167.0 [160.0; 175.0]
Body weight [kg]	54,793	78.5 [66.2; 93.6]	43,454	78.1 [65.9; 92.7]
Body Mass index [kg/m^2^]	54,793	27.8 [23.9; 32.7]	43,454	27.6 [23.8; 32.4]
Waist circumference [cm]	53,031	96.0 [85.0; 108.0]	42,084	95.5 [84.6; 107.0]
Pulse [beats per minute]	50,986	72.0 [64.0; 80.0]	40,968	72.0 [64.0; 80.0]
Systolic blood pressure [mm Hg]	46,156	117.0 [109.0; 128.0]	35,628	117.0 [109.0; 128.0]
Diastolic blood pressure [mm Hg]	46,105	72.0 [64.7; 79.0]	35,577	71.3 [64.0; 78.0]
White blood count [10^9^/L]	52,055	7.0 [5.7; 8.5]	41,316	7.0 [5.7; 8.5]
Hemoglobin [g/dL]	52,056	14.2 [13.1; 15.2]	41,317	14.2 [13.2; 15.3]
Hematocrit [%]	52,056	41.9 [38.9; 44.9]	41,317	41.9 [38.8; 45.0]
Platelets [10^9^/L]	52,055	250.0 [213.0; 294.0]	41,316	250.0 [213.0; 295.0]
C‐reactive protein (CRP) [mg/L]	42,722	0.5 [0.2; 1.7]	32,292	0.3 [0.1; 1.0]
Sodium [mmol/L]	47,133	139.0 [138.0; 141.0]	40,748	139.0 [138.0; 141.0]
Potassium [mmol/L]	47,122	4.0 [3.8; 4.2]	40,744	4.0 [3.8; 4.2]
Chloride [mmol/L]	47,132	103.0 [101.0; 105.0]	40,747	104.0 [102.0; 105.0]
Bicarbonate [mmol/L]	47,086	25.0 [23.0; 26.0]	40,704	25.0 [23.0; 26.0]
Blood urea nitrogen [mg/dL]	47,127	12.0 [9.0; 15.0]	40,745	12.0 [9.0; 15.0]
Creatinine [mg/dL]	47,131	0.8 [0.7; 1.0]	40,747	0.8 [0.7; 1.0]
Alanine aminotransferase [IU/L]	47,078	21.0 [15.0; 29.0]	40,696	21.0 [16.0; 29.0]
Aspartate aminotransferase [IU/L]	47,033	22.0 [18.0; 27.0]	40,679	22.0 [19.0; 27.0]
Alkaline phosphatase [IU/L]	43,137	69.0 [56.0; 84.0]	36,760	68.0 [55.0; 83.0]
Total bilirubin [mg/dL]	47,111	0.6 [0.4; 0.8]	40,727	0.6 [0.5; 0.8]
Albumin [g/dL]	47,135	4.3 [4.0; 4.5]	40,750	4.3 [4.0; 4.5]
Total protein [g/dL]	47,073	7.2 [6.9; 7.5]	40,691	7.2 [6.9; 7.5]
Gamma‐glutamyltransferase [U/L]	47,126	20.0 [14.0; 31.0]	40,744	20.0 [14.0; 31.0]
Hemoglobin A1c [%]	51,999	5.4 [5.1; 5.7]	41,288	5.4 [5.1; 5.7]
Fasting glucose [mg/dL]	25,514	98.0 [91.0; 107.0]	19,988	97.0 [90.1; 106.0]
Fasting insulin [μU/mL]	24,987	9.8 [6.2; 16.1]	19,620	9.8 [6.3; 16.0]
HOMA‐IR	24,950	2.4 [1.5; 4.2]	19,596	2.4 [1.5; 4.2]
Fasting triglycerides [mg/dL]	22,823	100.0 [68.0; 152.0]	19,658	102.0 [70.0; 155.0]
Fasting total cholesterol [mg/dL]	51,258	189.0 [163.0; 218.0]	40,851	190.0 [164.0; 219.0]
Fasting LDL cholesterol [mg/dL]	22,097	111.0 [89.0; 135.0]	18,961	112.0 [90.0; 136.0]
Fasting HDL cholesterol [mg/dL]	51,258	50.0 [41.0; 61.0]	40,851	50.0 [41.0; 61.0]
Median controlled attenuation parameter (CAP) [dB/m]	14,585	258.0 [214.0; 306.0]	3919	260.0 [214.0; 307.0]
Median liver stiffness [kPa]	14,588	5.0 [4.1; 6.2]	3920	4.9 [4.0; 6.1]
Truncal fat percentage [%]	27,482	32.4 [25.5; 39.4]	27,436	32.4 [25.5; 39.4]
Total fat percentage [%]	26,802	32.7 [26.3; 40.0]	26,756	32.7 [26.3; 40.0]
Lumbar spine bone mineral density [g/cm^2^]	27,492	1.0 [0.9; 1.1]	27,446	1.0 [0.9; 1.1]

*Note:* Values are presented as median and in brackets the first and third quartiles. The number of subjects for which data were available is indicated in the second and fourth column.

Abbreviations: CAP, Controlled Attenuation Parameter; CRP, C‐Reactive Protein; HDL, High‐Density Lipoprotein; HOMA‐IR, Homeostatic Model Assessment of Insulin Resistance; LDL, Low‐Density Lipoprotein.

Laboratory results included median hemoglobin A1c (5.4%; 5.4%), fasting glucose (98 mg/dL; 97 mg/dL) with fasting insulin (9.8 μU/mL; 9.8 μU/mL), and HOMA‐IR (2.4; 2.4). Fasting lipid labs include triglycerides (100.0 mg/dL; 102.0 mg/dL), total cholesterol (189.0 mg/dL; 190.0 mg/dL), low‐density lipoprotein (LDL, 112.0 mg/dL; 112.0 mg/dL), and high‐density lipoprotein (HDL, 50.0 mg/dL; 50.0 mg/dL). Total and truncal fat percentages were equal between both cohorts (32.7%; 32.4%). Liver imaging studies by Fibroscan provided controlled attenuation parameter (CAP) (258.0 dB/m; 260.0 dB/m) and liver stiffness (5.0 kPa; 4.9 kPa) (Table 1).

### 3.2 The Prevalence of Obesity Classes 4 and 5 Is Increasing, Particularly in Females and Non‐Hispanic Blacks

**FIGURE 1:**
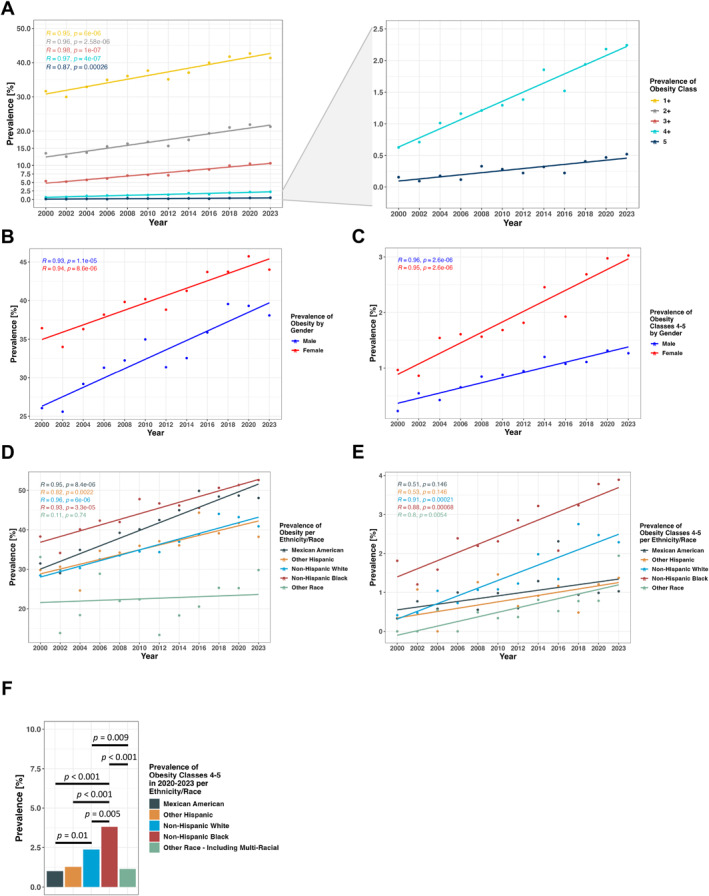
The prevalence of obesity classes 4 and 5 in adults is increasing and particularly affects females and non‐Hispanic Blacks. (A) Relative prevalence of adulthood obesity classes 1 +, 2 +, 3 +, and 4 +, and 5 from 2000–2023 (total, *n* = 54,793; class 1 +, *n* = 20,347, class 2 +, *n* = 9527; class 3+, *n* = 4296; class 4 +, *n* = 808; class 5; *n* = 158). (B) Relative prevalence of obesity in males and females from 2000 to 2023. (C) relative prevalence of obesity classes 4–5 in males and females from 2000 to 2023 (males; *n* = 26,113; females, *n* = 28,680). (D) Relative prevalence of obesity per ethnicity/race from 2000 to 2023. (E) relative prevalence of obesity classes 4–5 per ethnicity/race from 2000 to 2023 (Mexican American, *n* = 9995; other Hispanic, *n* = 5000; non‐Hispanic White, *n* = 21,164; non‐Hispanic Black, *n* = 12,370; other Race—including Multi‐Racial, *n* = 6264). (F) Relative prevalence of obesity classes per ethnicity/race from the combined 2020 to 2023 cohort (Mexican American, *n* = 1301; other Hispanic, *n* = 1256; non‐Hispanic White, *n* = 4382; non‐Hispanic Black, *n* = 2468; other Race—including Multi‐Racial, *n* = 1843). Statistical significance is indicated by *p* < 0.05.

From 2000 to 2023, the prevalence of overall obesity increased significantly. The prevalence of any obesity increased by 30.6% from 31.7% to 41.4%, class 2 and higher by 57.8% from 13.5% to 21.3, class 3 and higher by 97.0% from 5.4% to 10.6%, class 4 and higher by 255.6% from 0.6% to 2.2%, and class 5 by 246.7% from 0.2% to 0.5% (all values rounded to one decimal place after the comma; all *p* < 0.001) (Figure 1A). Female obesity increased from 36.4% in 2000 to 44.0% in 2023 (*R* = 0.94, *p* < 0.001) and male obesity from 26.1% to 38.1% (*R* = 0.93, *p* < 0.001) (Figure 1B). In classes 4 and 5, females increased from 0.9% to 3.0% (*R* = 0.95, *p* < 0.001) and males increased from 0.2% to 1.3% (*R* = 0.96, *p* < 0.001) (Figure 1C). Amongst ethnicities/races, non‐Hispanic Black had the highest prevalence in all obesity classes, rising from 38.3% in 2000 to 52.6% in 2023 (*R* = 0.93, *p* < 0.001).

Mexican Americans with the second highest overall prevalence increased from 31.8% to 48.1% (*R* = 0.95, *p* < 0.001). Comparatively, the prevalence of obesity in non‐Hispanic Whites increased from 28.5% to 40.9% (*R* = 0.96, *p* < 0.001) and other Hispanics increased from 29.8% to 38.2% (*R* = 0.82, *p* = 0.002); however, other races had an insignificant change (Figure 1D). The prevalence of classes 4 and 5 for non‐Hispanic Blacks rose from 1.8% to 3.9% and non‐Hispanic Whites from 0.4% to 2.3% (both *p* < 0.001). For Mexican Americans, there was an insignificant upward trend in Class 4 and 5 over time (*R* = 0.51, *p* = 0.15) (Figure 1E).

Overall, in 2020–2023, non‐Hispanic Blacks had the highest prevalence of classes 4 and 5 with 3.8%, which was significantly higher than any other ethnicity/race (*p* < 0.001 to *p* = 0.005 after adjustment for multiplicity). The second highest prevalence was among non‐Hispanic Whites at 2.4%, significantly higher than Mexican Americans or other Hispanics (1.0% and 1.3%, *p* = 0.01 and *p* = 0.009, respectively) (Figure 1F).

### 3.3 Adulthood Obesity Classes 4 and 5 Have the Highest Total and Truncal Fat Percentages but Also the Highest Bone Mineral Density Compared With Lower BMI Groups

Waist circumference, total fat percentage circumference, truncal fat percentage, and lumbar spine bone mineral density exhibited a close direct relationship with increasing BMI throughout the study population (Supporting Information S1: Figure S1). Classes 4 and 5 obesity demonstrated significantly higher waist circumference (145.0 and 156.0 cm vs. 88.2 cm, 106.0 cm, 116.0 cm, 128.0 cm; respectively), total fat percentage circumference (48.7% and 52.4% vs. 29.2%, 37.2%, 42.0%, 45.7%; respectively), truncal fat percentage (48.6% and 50.9% vs. 28.4%, 37.3%, 41.6%, 45.2%; respectively), and lumbar spine bone mineral density (1.1 g/cm^2^ and 1.3 g/cm^2^ vs. 1.0 g/cm^2^, 1.0 g/cm^2^, 1.1 g/cm^2^, 1.1 g/cm^2^; respectively) compared with no obesity or classes 1, 2, and 3 obesity in adulthood (all *p* < 0.001 after adjustment for multiple comparison, Table 2 and Supporting Information S1: Table S1).

**TABLE 2:** Comparison of obesity classes by physical exam, laboratory, and imaging data of the entire study population.

	No obesity	Class 1	Class 2	Class 3	Class 4	Class 5
	*n* = 34,446	*n* = 10,820	*n* = 5231	*n* = 3488	*n* = 650	*n* = 158
Body Mass index [kg/m^2^]	25.0 [22.4; 27.3]	32.1 [31.0; 33.4]	37.0 [35.9; 38.3]	43.1 [41.3; 45.5]	52.9 [51.2; 55.7]	64.7 [62.5; 68.2]
Waist circumference [cm]	88.2 [80.3; 95.5]	106.0 [101.0; 111.0]	116.0 [110.0; 122.0]	128.0 [121.0; 135.0]	145.0 [138.0; 153.0]	156.0 [148.0; 165.0]
Truncal fat percentage [%]	28.4 [22.5; 34.4]	37.3 [32.3; 42.2]	41.6 [37.1; 45.6]	45.2 [41.2; 49.1]	48.6 [45.2; 51.8]^5^	50.9 [45.7; 53.9]^4^
Total fat percentage [%]	29.2 [23.9; 36.0]	37.2 [30.8; 42.9]	42.0 [35.5; 45.9]	45.7 [39.8; 49.0]	48.7 [43.8; 51.6]	52.4 [44.4; 54.1]
Lumbar spine bone mineral density [g/cm^2^]	1.0 [0.9; 1.1]^1^	1.0 [0.9; 1.1]^0^	1.1 [1.0; 1.2]	1.1 [1.0; 1.2]	1.1 [1.0; 1.3]	1.3 [1.2; 1.4]
MASLD	997 (11.7%)	1143 (38.3%)	851 (52.9%)^5^	765 (65.2%)^5^	176 (80.0%)	34 (58.6%)^2,3^
Hepatic steatosis grade 1+	1014 (11.9%)	1143 (38.3%)	851 (52.9%)^5^	765 (65.2%)^5^	176 (80.0%)	34 (58.6%)^2,3^
Hepatic steatosis grade 2+	419 (4.9%)	625 (21.0%)	561 (34.9%)^5^	545 (46.4%)^5^	135 (61.4%)	25 (43.1%)^2,3^
Hepatic steatosis grade 3	337 (4.0%)	529 (17.7%)	491 (30.5%)^5^	509 (43.4%)^5^	131 (59.5%)	25 (43.1%)^2,3^
Hepatic fibrosis stage 2+	377 (4.4%)	280 (9.4%)	306 (19.0%)	378 (32.2%)	126 (57.0%)^5^	41 (70.7%)^4^
Hepatic fibrosis stage 3+	205 (2.4%)	173 (5.8%)	175 (10.9%)	277 (23.6%)	103 (46.6%)	38 (65.5%)
Hepatic fibrosis stage 4	91 (1.1%)	83 (2.8%)	70 (4.4%)	133 (11.3%)	58 (26.2%)	32 (55.2%)
Median controlled attenuation parameter (CAP) [dB/m]	230.0 [198.0; 270.0]	284.0 [247.0; 322.0]	306.0 [268.0; 346.0]	324.0 [286.0; 366.0]^5^	354.0 [311.0; 388.0]	321.0 [283.0; 374.0]^3^
Median liver stiffness [kPa]	4.6 [3.8; 5.6]	5.2 [4.3; 6.3]	5.5 [4.4; 7.3]	6.6 [5.1; 9.4]	8.9 [6.5; 14.1]	15.3 [7.7; 28.2]
Metabolic syndrome	5299 (38.1%)	3390 (70.2%)	1730 (76.0%)^5^	1247 (81.0%)^4,5^	204 (85.7%)^3,5^	35 (89.7%)^2,3,4^
Prediabetes	5284 (16.2%)	2770 (26.8%)	1533 (30.7%)	1141 (34.5%)^4^	229 (37.9%)^3^	72 (47.7%)
Diabetes	2168 (6.6%)	1605 (15.5%)	1036 (20.6%)	876 (26.2%)^5^	199 (32.6%)^5^	47 (30.7%)^3,4^
Hemoglobin A1c [%]	5.3 [5.1; 5.6]	5.5 [5.2; 5.8]	5.6 [5.3; 6.0]	5.7 [5.4; 6.2]	5.8 [5.4; 6.4]^5^	5.8 [5.5; 6.3]^4^
Fasting glucose [mg/dL]	96.0 [89.6; 103.0]	101.0 [94.0; 111.0]	102.0 [95.0; 115.0]	105.0 [96.0; 118.0]^5^	110.0 [99.0; 131.0]^5^	107.0 [96.0; 135.0]^3,4^
Fasting insulin [μU/mL]	7.6 [5.1; 11.4]	13.5 [9.3; 19.8]	17.3 [11.4; 25.7]	21.1 [14.4; 31.9]	27.4 [18.4; 39.1]^5^	26.2 [16.4; 39.3]^4^
HOMA‐IR	1.8 [1.2; 2.8]	3.5 [2.3; 5.4]	4.6 [2.9; 7.2]	5.8 [3.7; 9.4]	7.5 [4.9; 12.1]^5^	7.3 [4.4; 12.0]^4^
Insulin resistance	4933 (31.2%)	3415 (70.4%)	1901 (81.5%)	1427 (88.5%)	249 (97.3%)^5^	72 (97.3%)^4^
Severe insulin resistance	103 (0.7%)	156 (3.2%)	118 (5.1%)	137 (8.5%)^5^	39 (15.2%)^5^	9 (12.2%)^3,4^
Fasting triglycerides [mg/dL]	91.0 [63.0; 136.0]^5^	121.0 [81.0; 178.0]^2^	120.0 [82.0; 177.0]^1,3^	115.0 [81.0; 169.0]^2,4^	107.0 [75.5; 156.0]^3^	93.0 [72.5; 120.0]^0^
Fasting total cholesterol [mg/dL]	187.0 [162.0; 216.0]	195.0 [169.0; 224.0]	191.0 [166.0; 219.0]	186.0 [162.0; 212.0]	177.0 [154.0; 203.0]^5^	180.0 [155.0; 208.0]^4^
Fasting LDL cholesterol [mg/dL]	109.0 [87.0; 133.0]^5^	118.0 [95.0; 141.0]^5^	113.0 [92.0; 135.0]^3,5^	113.0 [90.0; 134.0]^2,5^	106.0 [85.0; 121.0]	115.0 [96.5; 138.0]^0,1,2,3^
Fasting HDL cholesterol [mg/dL]	53.0 [44.0; 65.0]	46.0 [39.0; 56.0]^4,5^	45.0 [38.0; 54.0]^3,4,5^	45.0 [39.0; 53.0]^2,4,5^	45.0 [38.0; 55.0]^1,2,3,5^	45.0 [39.0; 56.0]^1,2,3,4^
C‐reactive protein (CRP) [mg/L]	0.3 [0.1; 1.0]	0.8 [0.3; 2.5]	1.3 [0.5; 4.3]	2.3 [0.8; 6.3]	4.7 [1.4; 11.1]	6.1 [1.9; 14.3]
Pulse [beats per minute]	70.3 [64.0; 80.0]	72.0 [65.0; 80.0]	74.0 [67.0; 82.0]	76.0 [68.0; 84.0]	78.0 [69.0; 88.0]	84.0 [74.0; 92.0]
Systolic blood pressure [mm Hg]	115.0 [107.0; 126.0]	120.0 [111.0; 131.0]^5^	121.0 [112.0; 132.0]^3,4,5^	122.0 [112.0; 133.0]^2,4,5^	123.0 [114.0; 133.0]^2,3,5^	123.0 [112.0; 132.0]^1,2,3,4^
Diastolic blood pressure [mm Hg]	70.3 [63.3; 77.3]	74.0 [66.7; 81.3]	74.7 [67.3; 82.0]^3,4^	75.3 [67.7; 83.3]^2^	75.7 [68.7; 83.3]^2^	78.8 [69.6; 86.0]

*Note:* Every group is significantly different from each other group after pairwise Wilcoxon–Whitney–Mann rank‐sum tests with False Discovery Rate (FDR) correction for multiplicity; not significant adjusted comparisons with a specific class are indicated by a superscript number of that class in the respective column, where ^“0”^ (zero) equals no obesity. *p* value < 0.05 indicates statistical significance. Exact *p* values are shown in Supporting Information S1: Table S1. Values are presented as median and in brackets the first and third quartiles.

Abbreviations: CAP, Controlled Attenuation Parameter; CRP, C‐Reactive Protein; HDL, High‐Density Lipoprotein; HOMA‐IR, Homeostatic Model Assessment of Insulin Resistance; LDL, Low‐Density Lipoprotein; MASLD, Metabolic Dysfunction‐Associated Steatotic Liver Disease.

### 3.4 Extremely Severe Adulthood Obesity Is Associated With Higher Prevalence and Risk of MASLD and Advanced Fibrosis

There is a well‐documented relationship between MASLD and obesity in adulthood [23]. The prevalence of MASLD is lowest among those without obesity (11.7%) and increases with higher classes (Table 2 and Supporting Information S1: Table S1). However, the MASLD prevalence peaks at class 4 with 80% and is significantly lower in class 5 with 58.6%. A similar pattern occurs with CAP measurements. The lowest median CAP is the group without obesity (230.0 dB/m) and successively increases with BMIs (Supporting Information S1: Figure S2A) with the highest values in class 4 (354.0 dB/m) and significantly lower in class 5 (321.0 dB/m).

Similarly, the prevalence of the hepatic steatosis severities increased with worsening obesity—the highest in class 4 and lower in class 5 (Table 2 and Supporting Information S1: Table S1). When stratified by combined obesity classes, extremely severe obesity (classes 4–5) demonstrated a significantly higher MASLD prevalence with 75.5% than the group without obesity (11.7%) or obesity classes 1–3 (47.9%) (Supporting Information S1: Table S2). Liver stiffness measurement exhibited a positive association with BMI (*R* = 0.39, *p* < 0.001) (Supporting Information S1: Figure S2B). Comparing all different BMI groups, obesity class 5 was associated with the highest median liver stiffness (15.3 vs. 4.6 kPa with no obesity and 5.2 kPa in class 1 obesity), highest prevalence of hepatic fibrosis stages 2–4, 3–4, and 4 (70.7%, 65.5%, 55.2% vs. 4.4%, 2.4%, 1.1% without obesity or 9.4%, 5.8%, 2.8% in class 1 obesity, respectively, all *p* < 0.001) (Table 2 and Supporting Information S1: Table S1).

Higher obesity classes were associated with a higher likelihood of liver disease (Supporting Information S1: Tables S3 and S4). The odds ratio for MASLD in extremely severe obesity or classes 1–3 was 23.3 [95% confidence interval (CI), 17.6; 30.9] or 6.9 [95% CI, 6.4; 7.5] compared with the group without obesity (Supporting Information S1: Table S3). The odds ratio for hepatic fibrosis stages 2–4, 3–4, and 4 were highest in obesity class 5 with 52.2 [95% CI, 29.4; 92.8], 77.3 [95% CI, 44.2; 135.0], and 114.0 [95% CI, 65.5; 200.0] compared with the group with no obesity (Supporting Information S1: Table S4).

### 3.5 Adulthood Obesity Classes 4 and 5 Are Associated With Prediabetes, Type 2 Diabetes Mellitus, and Severe Insulin Resistance

Obesity is strongly linked to T2DM [24]. Those with higher BMI had higher Hemoglobin A1c (HbA1c) (*R* = 0.25, *p* < 0.001) (Supporting Information S1: Figure S2C), fasting glucose levels (*R* = 0.24, *p* < 0.001) (Supporting Information S1: Figure S2D), fasting insulin levels (*R* = 0.55, *p* < 0.001) (Supporting Information S1: Figure S2E), and Homeostatic Model Assessment of Insulin Resistance (HOMA‐IR) (*R* = 0.56, *p* < 0.001) (Supporting Information S1: Figure S2F). Hence, the prevalence of prediabetes and T2DM was highest in obesity classes 4 (37.9% and 32.6%) and 5 (47.7% and 30.7%) compared with the group without obesity (16.2% and 6.6%) (Table 2). Classes 4 and 5 had a median HbA1c 5.8%, which was significantly higher than those without obesity (5.3%) and classes 1–3 (5.5%). Classes 4 and 5 also had a higher median fasting glucose (110 [mg/dL] vs. 96 [mg/dL] and 102 [mg/dL]; all *p* < 0.001) and median fasting insulin (27.4 μU/mL, 7.6 μU/mL and 15.7 μU/mL; all *p* < 0.001) (Supporting Information S1: Table S2).

The prevalence of insulin resistance and severe insulin resistance was significantly higher in obesity classes 4–5 than without obesity or in classes 1–3 (97.3% and 14.5% vs. 31.2% and 0.7% as well as 76.6% and 4.7%, respectively; all *p* < 0.001) (Supporting Information S1: Table S2). Thus, classes 4‐5 had significantly higher odds ratios than classes 1–3 of having prediabetes, T2DM, insulin resistance, and severe insulin resistance (3.4 [95% CI, 3.0; 4.0], 6.7 [95% CI, 5.7; 7.9], 78.7 [95% CI, 40.6; 153], 25.9 [95% CI, 18.0; 37.2] vs. 2.1 [95% CI, 2.1; 2.2], 3.3 [95% CI, 3.1; 3.5], 7.2 [95% CI, 6.8; 7.7], 7.5 [95% CI, 6.0; 9.3], respectively; with the group without obesity as the reference) (Supporting Information S1: Table S3).

### 3.6 Extremely Severe Adulthood Obesity Is Linked to Other Cardiometabolic Risk Factors Including Metabolic Syndrome

Metabolic syndrome is a condition characterized by metabolic dysregulations that increases the risk of developing T2DM and cardiovascular diseases [24, 25]. The prevalence of metabolic syndrome increased with obesity severity and was highest in class 5 obesity with 89.7% and lowest with 38.1% in the group without obesity (Table 2 and Supporting Information S1: Table S1). Obesity classes 4 and 5 had a significantly higher odds ratio of having metabolic syndrome than classes 1–3 (10.2 [95% CI, 7.2; 14.4] and 4.5 [95% CI, 4.3; 4.8]; with the group without obesity as the reference) (Supporting Information S1: Table S3). The lipid fractions were most abnormal with class 1 obesity and were closer to normal range with more severe obesity (Supporting Information S1: Figure S3, Table 2, Supporting Information S1: Table S1, and S2): Fasting triglyceride, total cholesterol, and LDL cholesterol were significantly increased with class 1 obesity versus no obesity (121.0 mg/dL vs. 91.0 mg/dL, 195.0 mg/dL vs. 187.0 mg/dL, 118.0 mg/dL vs. 109.0 mg/dL, respectively), and were lower with obesity classes 4 and 5 (107.0 mg/dL and 93.0 mg/dL, 177.0 mg/dL and 180.0 mg/dL, 106.0 mg/dL and 115.0 mg/dL, respectively), whereas HDL cholesterol was significantly decreased with class 1 obesity versus no obesity (46.0 mg/dL vs. 53.0 mg/dL) and remained low with classes 4 and 5 (45.0 mg/dL and 45.0 mg/dL) (Table 2).

As obesity is associated with subclinical systemic inflammation [26], serum C‐reactive protein (CRP) levels were assessed and CRP correlated with BMI (*R* = 0.43, *p* < 0.001) (Supporting Information S1: Figure S3E). The median CRP levels in the group without obesity, classes 1–3, and 4–5 were 0.3 mg/L, 1.1 mg/L, and 4.7 mg/L, respectively (Supporting Information S1: Table S2). Pulse, systolic and diastolic blood pressure all correlated positively with BMI (*R* = 0.12, *R* = 0.18, *R* = 0.19; all *p* < 0.001) (Supporting Information S1: Figure S4). Pulse, systolic and diastolic blood pressure were all significantly higher in obesity classes 4 and 5 versus no obesity and classes 1–3 (80.0 bpm vs. 70.3 bpm vs. 74.0 bpm; 123.0 vs. 115.0 mm Hg vs. 121.0 mm Hg; 76.0 vs. 70.3 mm Hg vs. 74.3 mm Hg, respectively) (Supporting Information S1: Table S2).

### 3.7 Adulthood Obesity Classes 4 and 5 Have Higher Rates of Mortality Over Time Compared With Those Without Obesity and Classes 1 to 3

**FIGURE 2:**
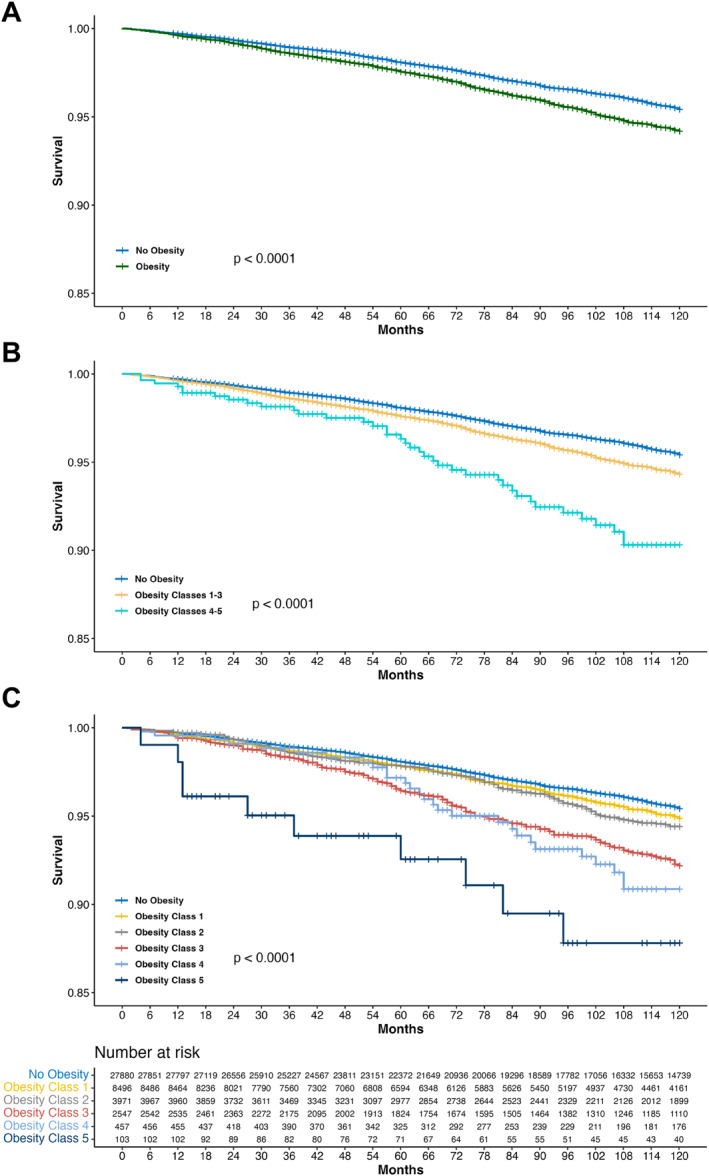
Obesity classes 4–5 are associated with higher mortality compared with obesity classes 1–3 or no obesity. (A) Kaplan–Meier survival curves for cohorts with obesity versus without obesity over 120 months (total, *n* = 43,454; obesity, *n* = 15,574; without obesity, *n* = 27,880). (B) Kaplan–Meier survival curves for cohorts with obesity classes 4–5 versus obesity classes 1–3 versus without obesity over 120 months (without obesity, *n* = 27,880; classes 1–3; *n* = 15,014; classes 4–5, *n* = 560). (C) Kaplan–Meier survival curves for cohorts with obesity classes 1, 2, 3, 4, 5, and without obesity over 120 months (without obesity, *n* = 27,880; class 1, *n* = 8496; class 2, *n* = 3971; class 3, *n* = 2547; class 4, *n* = 457; class 5, *n* = 103). Statistical significance is indicated by *p* < 0.05.

Obesity and higher obesity severity were associated with increased mortality (Figure 2, Table 3): Any obesity was associated with significantly increased risk for mortality over 10 years with a hazard ratio (HR) of 1.3 (95% CI, 1.2–1.4) compared with participants without obesity (Figure 2A, Table 3). Furthermore, obesity classes 4–5 and 1–3 were associated with a higher 10‐year mortality risk than the group without obesity (HR of 2.2, 95% CI 1.6–3.0, *p* < 0.001; HR of 1.3, 95% CI 1.1–1.4, *p* < 0.001; respectively) (Figure 2B, Table 3). The 10‐year mortality HR of obesity classes 4–5 versus 1–3 was 1.8 (95% CI 1.3–2.4, *p* < 0.001). Finally, obesity classes 1, 2, 3, 4, and 5 had a 13%, 22%, 75%, 97%, and 220% higher mortality hazard, respectively, over the 10‐year observation period compared with subjects without obesity (HR of 1.1, 95% CI 1.0–1.3; HR of 1.2, 95% CI 1.0–1.4; HR of 1.8, 95% CI 1.5–2.1; HR of 2.0, 95% CI 1.4–2.9; HR of 3.2, 95% CI 1.7–6.0; respectively) (Figure 2C, Table 3; see Table 3 for additional hazard ratios for 1‐year, 3‐year, and 5‐year mortality).

**TABLE 3:** Hazard ratio of mortality per univariate cox regression per obesity severity.

	1‐year mortality			3‐year mortality			5‐year mortality			10‐year mortality		
	HR	95% CI	*p* value	HR	95% CI	*p* value	HR	95% CI	*p* value	HR	95% CI	*p* value
Obesity versus no obesity	1.4	1.0–1.9	0.07	1.3	1.1–1.6	0.002	1.3	1.1–1.5	< 0.001	1.3	1.2–1.4	< 0.001
Obesity classes 1–3 versus no obesity	1.3	1.0–1.8	0.1	1.3	1.1–1.6	0.003	1.3	1.1–1.5	0.001	1.3	1.1–1.4	< 0.001
Obesity classes 4–5 versus no obesity	2.4	0.9–6.6	0.09	1.8	1.0–3.3	0.07	1.9	1.2–3.1	0.006	2.2	1.6–3.0	< 0.001
Obesity class 1 versus no obesity	1.3	0.9–2.0	0.20	1.2	1.0–1.5	0.07	1.2	1.0–1.4	0.12	1.1	1.0–1.3	0.05
Obesity class 2 versus no obesity	0.9	0.5–1.8	0.82	1.3	1.0–1.7	0.08	1.1	0.9–1.5	0.28	1.2	1.0–1.4	0.02
Obesity class 3 versus no obesity	2.0	1.1–3.4	0.01	1.6	1.1–2.2	0.006	1.8	1.5–2.3	< 0.001	1.8	1.5–2.1	< 0.001
Obesity class 4 versus no obesity	1.5	0.4–6.0	0.59	1.1	0.5–2.6	0.86	1.4	0.8–2.6	0.24	2.0	1.4–2.9	< 0.001
Obesity class 5 versus no obesity	6.6	1.6–26.7	0.009	5.0	2.1–12.1	< 0.001	4.2	2.0–8.9	< 0.001	3.2	1.7–6.0	< 0.001

Abbreviations: CI, Confidence Interval; HR, Hazard Ratio.

## 4 Discussion

In 2023, the prevalence of obesity class 4 and higher was 2.2% and class 5 was 0.5% among US adults, representing increases of 255.6% and 246.7%, respectively, since 2000. This increase is consistent with models that predict the prevalence of class 3 obesity to be greater than 25% in 25 states by 2030 [3]. Extremely severe obesity incurs a significantly higher risk of comorbidities than those with lower obesity classes. For instance, classes 4 and 5 have 236% higher odds of MASLD, 812% higher odds of liver fibrosis stage 4 or cirrhosis, 106% higher odds of T2DM, 987% higher odds of insulin resistance, 247% higher odds of severe insulin resistance, and 125% higher risk of metabolic syndrome than those with obesity classes 1–3 (Supporting Information S1: Table S3).

These findings are significant considering that liver steatosis and fibrosis can progress to cirrhosis and hepatocellular carcinoma [27, 28]. Notably, the prevalence of hepatic steatosis and MASLD is highest in class 4 but lower in class 5 (Table 2 and Supporting Information S1: Table S1). This could possibly be explained by the presence of burnt‐out MASH in class 5, where hepatic fat decreases sharply with advanced fibrosis/cirrhosis [29, 30, 31].

Furthermore, classes 4 and 5 have lower levels of fasting LDL cholesterol, total cholesterol, and triglycerides than classes 1–3. Although the mechanism underlying this pattern warrants further investigation, one possible explanation for the decrease in serum and hepatic lipid content in class 5 may involve the relationship between obesity and chronic inflammation [26, 32]. CRP levels increase with BMI and are highest in class 5 obesity (Table 2), which may potentially contribute to increased induction of lipid catabolism and fibrosis in the liver, though confirmatory mechanistic studies are needed [33]. Although this may initially result in an atherogenic serum lipid profile, once the cirrhosis or the burnt‐out MASH stage is reached, the decreased amount of hepatic fat may contribute to the lower serum LDL cholesterol, total cholesterol, and triglyceride levels in cirrhosis [34, 35, 36, 37]. This may explain the observed liver and serum lipid profiles in obesity class 5 in this cohort.

The study also shows that extremely severe obesity is associated with an increased risk of metabolic syndrome with concurrent increased measures of blood pressure, waist circumference, and truncal fat percentage in classes 4 and 5 compared with classes 1–3. Metabolic syndrome is characterized by metabolic dysregulations that increase the risk of developing T2DM and cardiovascular diseases [24, 25] and even mortality from cardiovascular disease and all‐cause mortality, underscoring the importance of comprehensive risk factor management [5, 38].

Extremely severe obesity has an increased risk of severe insulin resistance, prediabetes, and T2DM. Obesity and T2DM are strongly associated, such that an important part of diabetes management is weight loss [39]. Adipose tissue releases adipokines and induces inflammation amongst other diabetogenic factors that are suspected to contribute to insulin resistance [33, 40].

These data highlight the demographics most affected by extremely severe obesity. Females have a higher prevalence of extremely severe obesity than males, and non‐Hispanic Blacks have the highest prevalence of class 4 and 5 obesity, which is in line with published data on obesity [41, 42, 43]. The rising evidence of racial and ethnic disparities in obesity warrants focus on targeted health interventions.

Immediate interventions against obesity are crucial as the prevalence of extremely severe obesity continues to rise. Metabolic and bariatric surgery represents the most effective and durable treatment for patients with classes 4 and 5 obesity and should be considered the preferred therapeutic modality for this population [11]. In the setting of a national shortage of weight loss medications, the authors suggest that the newest and most potent pharmacologic agents, including GLP‐1 and GLP‐1/GIP dual receptor agonists, should ideally be prioritized for the metabolically sickest patients [44, 45]. Patients with extremely severe obesity exactly represent such a population, given the dramatically elevated cardiometabolic comorbidity burden and mortality risk demonstrated in this analysis. Importantly, however, all patients with obesity—particularly those with metabolic comorbidities—should be evaluated and considered for anti‐obesity pharmacotherapy in accordance with clinical guidelines, regardless of obesity class. Based on the mortality data collected in this study, subjects with obesity, particularly those in classes 4 and 5, had a significantly worse survival than those without obesity. The higher prevalence of metabolic complications in extremely severe obesity (Table 2, Supporting Information S1: Table S1, and S2) likely contributes to the worse survival in this group versus no or milder forms of obesity (Figure 2 and Table 3). This is in line with prior evidence that obesity is associated with higher mortality [14].

The strengths of this study include usage of a national database comprising 54,793 individuals from diverse racial groups from 2000 to 2023. Another strength is the inclusion of mortality data from most of the participants (43,454 individuals). This study is the first to explore in detail the prevalence, comorbidities, and mortality of obesity classes 4 and 5 in adults with data supporting the necessity of immediate intervention against the rising obesity crisis. Limitations of the study include the use of liver imaging rather than biopsy to diagnose MASLD. However, performing a liver biopsy on every participant would have been unethical and therefore not feasible.

Future research should investigate the physiologic mechanisms behind the study's finding of class 5 having a lower prevalence of MASLD and insulin resistance than class 4. Additionally, further exploration into the factors (i.e., genetic, socioeconomic, psychosocial) that could contribute to extremely severe obesity in females and non‐Hispanic Blacks can aid in developing targeted interventions for groups most at risk.

In conclusion, this study demonstrates that extremely severe obesity is increasing in prevalence and is associated with significant metabolic complications and a high risk of mortality. Targeted public health interventions are hence required to support and help this patient population. Further, randomized controlled trials designed specifically for subjects with classes 4 and 5 obesity are necessary and will help to elucidate how those patients can be managed most effectively.

## Author Contributions

K.T. directly accessed and verified the underlying data reported in the manuscript, analyzed, and interpreted the data, and wrote and revised the manuscript. X.Z. directly accessed and verified the underlying data reported in the manuscript, provided statistical assistance, and edited the manuscript. P.H. designed the study, developed the method, supervised the work, directly accessed and verified the underlying data reported in the manuscript, analyzed, and interpreted the data, edited the manuscript, and is the guarantor of the study. All authors have approved the submitted version. P.H. affirms that the manuscript is an honest, accurate, and transparent account of the study being reported, that no important aspects of the study have been omitted, and that any discrepancies from the study as planned have been explained.

## Funding

The funders of the study had no role in study design, data collection, data analysis, data interpretation, or writing of the report. The authors were independent of the funders.

## Ethics Statement

The authors have nothing to report.

## Consent

The publicly available dataset was obtained from https://www.cdc.gov/nchs/nhanes [15].

## Conflicts of Interest

The authors declare no conflicts of interest.

## Supporting information


Supporting Information S1

## Data Availability

The raw data are available at https://www.cdc.gov/nchs/nhanes [15].
