# Supplementary material for: Prevalence, Comorbidities, and Mortality of Obesity Classes 4 and 5 in Adults
Source: Obes Sci Pract. 2026 Jul 22;12(4):e70171. doi: 10.1002/osp4.70171 (PMC13391446; doi:10.1002/osp4.70171)
Supplement: Supplementary file 1 — Supporting Information S1 [file OSP4-12-e70171-s001.pdf]

Prevalence, comorbidities, and mortality of obesity classes 4 and 5 in adults

Katelynn Tran, Xinlian Zhang, Phillipp Hartmann

Table of Contents

***Supplementary Tables and Figure*……………………………………………………………………………………2**
***Supplementary Tables*…………………………………………………………………………………………………2**
***Table S1*……2**
***Table S2*……6**
***Table S3*……9**
***Table S4*………10**
***Supplementary Figures*……………………………………………………………………………………………… 11**
***Figure S1*………11**
***Figure S2*………12**
***Figure S3*………14**
***Figure S4*………16**

Supplementary Tables and Figure

Supplementary Tables

Table S1. Detailed comparison of obesity classes by physical exam, laboratory, and imaging data of the entire study population.

	n	No Obesity ("0")	Class 1	Class 2	Class 3	Class 4	Class 5	P value overall	All pairwise P values <0.001 except
		n=34,446	*n=10,820*	*n=5,231*	*n=3,488*	*n=650*	*n=158*		
**Body Mass Index [kg/m²]**	54,793	25.0 [22.4;27.3]	32.1 [31.0;33.4]	37.0 [35.9;38.3]	43.1 [41.3;45.5]	52.9 [51.2;55.7]	64.7 [62.5;68.2]	<0.001	-
**Waist Circumference [cm]**	53,031	88.2 [80.3;95.5]	106.0 [101.0;111.0]	116.0 [110.0;122.0]	128.0 [121.0;135.0]	145.0 [138.0;153.0]	156.0 [148.0;165.0]	<0.001	-
**Truncal Fat Percentage [%]**	27,482	28.4 [22.5;34.4]	37.3 [32.3;42.2]	41.6 [37.1;45.6]	45.2 [41.2;49.1]	48.6 [45.2;51.8]	50.9 [45.7;53.9]	<0.001	4 vs 5: 0.064
**Total Fat Percentage [%]**	26,802	29.2 [23.9;36.0]	37.2 [30.8;42.9]	42.0 [35.5;45.9]	45.7 [39.8;49.0]	48.7 [43.8;51.6]	52.4 [44.4;54.1]	<0.001	4 vs 5: 0.014
**Lumbar Spine Bone Mineral Density [g/cm²]**	27,492	1.03 [0.94;1.13]	1.03 [0.94;1.14]	1.05 [0.95;1.16]	1.08 [0.98;1.20]	1.14 [1.01;1.27]	1.27 [1.15;1.36]	<0.001	0 vs 1: 0.988
**MASLD**	14,568							<0.001	1 vs 5: 0.003, 2 vs 5: 0.468, 3 vs 5: 0.407, 4 vs 5: 0.002
**Absent**		7,528 (88.3%)	1,839 (61.7%)	758 (47.1%)	409 (34.8%)	44 (20.0%)	24 (41.4%)		
**Present**		997 (11.7%)	1143 (38.3%)	851 (52.9%)	765 (65.2%)	176 (80.0%)	34 (58.6%)		
**Hepatic Steatosis Grade 1+**	14,585							<0.001	1 vs 5: 0.003, 2 vs 5: 0.468, 3 vs 5: 0.407, 4 vs 5: 0.002
**Absent**		7,528 (88.1%)	1,839 (61.7%)	758 (47.1%)	409 (34.8%)	44 (20.0%)	24 (41.4%)		

**Present**		1,014 (11.9%)	1,143 (38.3%)	851 (52.9%)	765 (65.2%)	176 (80.0%)	34 (58.6%)		
**Hepatic Steatosis Grade 2+**	14,585							<0.001	2 vs 5: 0.268, 3 vs 5: 0.719, 4 vs 5: 0.021
**Absent**		8,123 (95.1%)	2,357 (79.0%)	1,048 (65.1%)	629 (53.6%)	85 (38.6%)	33 (56.9%)		
**Present**		419 (4.91%)	625 (21.0%)	561 (34.9%)	545 (46.4%)	135 (61.4%)	25 (43.1%)		
**Hepatic Steatosis Grade 3**	14,585							<0.001	2 vs 5: 0.063, 3 vs 5: 1.000, 4 vs 5: 0.042
**Absent**		8,205 (96.1%)	2,453 (82.3%)	1,118 (69.5%)	665 (56.6%)	89 (40.5%)	33 (56.9%)		
**Present**		337 (3.95%)	529 (17.7%)	491 (30.5%)	509 (43.4%)	131 (59.5%)	25 (43.1%)		
**Hepatic Fibrosis Stage 2+**	14,588							<0.001	4 vs 5: 0.082
**Absent**		8,165 (95.6%)	2,704 (90.6%)	1,303 (81.0%)	796 (67.8%)	95 (43.0%)	17 (29.3%)		
**Present**		377 (4.4%)	280 (9.4%)	306 (19.0%)	378 (32.2%)	126 (57.0%)	41 (70.7%)		
**Hepatic Fibrosis Stage 3+**	14,588							<0.001	4 vs 5: 0.016
**Absent**		8,337 (97.6%)	2,811 (94.2%)	1,434 (89.1%)	897 (76.4%)	118 (53.4%)	20 (34.5%)		
**Present**		205 (2.4%)	173 (5.8%)	175 (10.9%)	277 (23.6%)	103 (46.6%)	38 (65.5%)		
**Hepatic Fibrosis Stage 4**	14,588							<0.001	1 vs 2: 0.006
**Absent**		8,451 (98.9%)	2,901 (97.2%)	1,539 (95.6%)	1,041 (88.7%)	163 (73.8%)	26 (44.8%)		
**Present**		91 (1.1%)	83 (2.8%)	70 (4.4%)	133 (11.3%)	58 (26.2%)	32 (55.2%)		
**Median Controlled Attenuation Parameter (CAP) [dB/m]**	14,585	230.0 [198.0;270.0]	284.0 [247.0;322.0]	306.0 [268.0;346.0]	324.0 [286.0;366.0]	354.0 [311.0;388.0]	321.0 [283.0;374.0]	<0.001	2 vs 5: 0.016, 3 vs 5: 0.855, 4 vs 5: 0.014
**Median Liver Stiffness [kPa]**	14,588	4.6 [3.8;5.6]	5.2 [4.3;6.3]	5.5 [4.4;7.3]	6.6 [5.1;9.4]	8.9 [6.5;14.1]	15.3 [7.7;28.2]	<0.001	-

**Metabolic Syndrome**	22,829							<0.001	1 vs 5: 0.018, 2 vs 3: 0.001, 2 vs 4: 0.001, 2 vs 5: 0.088, 3 vs 4: 0.110, 3 vs 5: 0.256, 4 vs 5: 0.669
**Absent**		8,610 (61.9%)	1,437 (29.8%)	546 (24.0%)	293 (19.0%)	34 (14.3%)	4 (10.3%)		
**Present**		5,299 (38.1%)	3,390 (70.2%)	1,730 (76.0%)	1,247 (81.0%)	204 (85.7%)	35 (89.7%)		
**Prediabetes**	52,033							<0.001	2 vs 3: 0.001, 2 vs 4: 0.001, 3 vs 4: 0.117, 3 vs 5: 0.001, 4 vs 5: 0.037
**Absent**		27,355 (83.8%)	7,567 (73.2%)	3,456 (69.3%)	2,171 (65.5%)	376 (62.1%)	79 (52.3%)		
**Present**		5,284 (16.2%)	2,770 (26.8%)	1,533 (30.7%)	1,141 (34.5%)	229 (37.9%)	72 (47.7%)		
**Diabetes**	52,193							<0.001	2 vs 5: 0.004, 3 vs 4: 0.002, 3 vs 5: 0.275, 4 vs 5: 0.723
**Absent**		30,540 (93.4%)	8,761 (84.5%)	3,982 (79.4%)	2,462 (73.8%)	411 (67.4%)	106 (69.3%)		
**Present**		2,168 (6.63%)	1,605 (15.5%)	1,036 (20.6%)	876 (26.2%)	199 (32.6%)	47 (30.7%)		
**Hemoglobin A1c [%]**	51,999	5.3 [5.1;5.6]	5.5 [5.2;5.8]	5.6 [5.3;6.0]	5.7 [5.4;6.2]	5.8 [5.4;6.4]	5.8 [5.5;6.3]	<0.001	3 vs 5: 0.045, 4 vs 5: 0.961
**Fasting Glucose [mg/dL]**	25,514	96.0 [89.6;103.0]	101.0 [94.0;111.0]	102.0 [95.0;115.0]	105.0 [96.0;118.0]	110.0 [99.0;131.0]	107.0 [96.0;135.0]	<0.001	1 vs 5: 0.002, 2 vs 5: 0.035, 3 vs 5: 0.255, 4 vs 5: 0.427
**Fasting Insulin [μU/mL]**	24,987	7.6 [5.12;11.4]	13.5 [9.27;19.8]	17.3 [11.4;25.7]	21.1 [14.4;31.9]	27.4 [18.4;39.1]	26.2 [16.4;39.3]	<0.001	3 vs 5: 0.009, 4 vs 5: 0.707
**HOMA-IR**	24,950	1.8 [1.2;2.8]	3.5 [2.3;5.4]	4.6 [2.9;7.2]	5.8 [3.7;9.4]	7.5 [4.9;12.1]	7.3 [4.4;12.0]	<0.001	3 vs 5: 0.010, 4 vs 5: 0.590

**Insulin Resistance**	24,950							<0.001	2 vs 5: 0.001, 3 vs 5: 0.032, 4 vs 5: 1.000
**Absent**		10,888 (68.8%)	1,438 (29.6%)	432 (18.5%)	186 (11.5%)	7 (2.7%)	2 (2.7%)		
**Present**		4,933 (31.2%)	3,415 (70.4%)	1,901 (81.5%)	1,427 (88.5%)	249 (97.3%)	72 (97.3%)		
**Severe insulin resistance**	24,987							<0.001	1 vs 5: 0.001, 2 vs 5: 0.016, 3 vs 4: 0.001, 3 vs 5: 0.401, 4 vs 5: 0.645
**Absent**		15,738 (99.3%)	4,710 (96.8%)	2,217 (94.9%)	1,477 (91.5%)	218 (84.8%)	65 (87.8%)		
**Present**		103 (0.7%)	156 (3.2%)	118 (5.1%)	137 (8.5%)	39 (15.2%)	9 (12.2%)		
**Fasting Triglycerides [mg/dL]**	22,823	91.0 [63.0;136.0]	121.0 [81.0;178.0]	120.0 [82.0;177.0]	115.0 [81.0;169.0]	107.0 [75.5;156.0]	93.0 [72.5;120.0]	<0.001	0 vs 5: 0.851, 1 vs 2: 0.851, 1 vs 3: 0.021, 1 vs 4: 0.011, 2 vs 3: 0.058, 2 vs 4: 0.013, 3 vs 4: 0.103, 4 vs 5: 0.017
**Fasting Total Cholesterol [mg/dL]**	51,258	187.0 [162.0;216.0]	195.0 [169.0;224.0]	191.0 [166.0;219.0]	186.0 [162.0;212.0]	177.0 [154.0;203.0]	180.0 [155.0;208.0]	<0.001	0 vs 3: 0.039, 0 vs 5: 0.019, 2 vs 5: 0.001, 3 vs 5: 0.046, 4 vs 5: 0.489
**Fasting LDL Cholesterol [mg/dL]**	22,097	109.0 [87.0;133.0]	118.0 [95.0;141.0]	113.0 [92.0;135.0]	113.0 [90.0;134.0]	106.0 [85.0;121.0]	115.0 [96.5;138.0]	<0.001	0 vs 3: 0.034, 0 vs 4: 0.041, 0 vs 5: 0.189, 1 vs 5: 0.861, 2 vs 3: 0.242, 2 vs 5: 0.530, 3 vs 4: 0.003, 3 vs 5: 0.374, 4 vs 5: 0.032

**Fasting HDL Cholesterol [mg/dL]**	51,258	53.0 [44.0;65.0]	46.0 [39.0;56.0]	45.0 [38.0;54.0]	45.0 [39.0;53.0]	45.0 [38.0;55.0]	45.0 [39.0;56.0]	<0.001	1 vs 4: 0.157, 1 vs 5: 0.748, 2 vs 3: 0.748, 2 vs 4: 0.748, 2 vs 5: 0.748, 3 vs 4: 0.748, 3 vs 5: 0.748, 4 vs 5: 0.748
**C-Reactive Protein (CRP) [mg/L]**	42,722	0.3 [0.1;1.0]	0.8 [0.3;2.5]	1.3 [0.5;4.3]	2.3 [0.8;6.3]	4.7 [1.4;11.1]	6.1 [1.9;14.3]	<0.001	4 vs 5: 0.028
**Pulse [beats per minute]**	50,986	70.3 [64.0;80.0]	72.0 [65.0;80.0]	74.0 [67.0;82.0]	76.0 [68.0;84.0]	78.0 [69.0;88.0]	84.0 [74.0;92.0]	<0.001	4 vs 5: 0.001
**Systolic Blood Pressure [mm Hg]**	46,156	115.0 [107.0;126.0]	120.0 [111.0;131.0]	121.0 [112.0;132.0]	122.0 [112.0;133.0]	123.0 [114.0;133.0]	123.0 [112.0;132.0]	<0.001	1 vs 4: 0.011, 1 vs 5: 0.465, 2 vs 3: 0.435, 2 vs 4: 0.435, 2 vs 5: 0.905, 3 vs 4: 0.771, 3 vs 5: 0.980, 4 vs 5: 0.905
**Diastolic Blood Pressure [mm Hg]**	46,105	70.3 [63.3;77.3]	74.0 [66.7;81.3]	74.7 [67.3;82.0]	75.3 [67.7;83.3]	75.7 [68.7;83.3]	78.8 [69.6;86.0]	<0.001	1 vs 4: 0.001, 2 vs 3: 0.045, 2 vs 4: 0.077, 2 vs 5: 0.004, 3 vs 4: 0.520, 3 vs 5: 0.022, 4 vs 5: 0.045

All adjusted p values are <0.001 after pairwise Wilcoxon-Whitney-Mann rank-sum tests with False Discovery Rate (FDR) correction for multiplicity, unless shown differently in the last column above, where “0” (zero) equals the no obesity group. P value < 0.05 indicates statistical significance. Values are presented as median and in brackets the first and third quartiles. The number of subjects for which data were available is indicated in the second column. CAP, Controlled Attenuation Parameter; CRP, C-Reactive Protein; HDL, High-Density Lipoprotein; HOMA-IR, Homeostatic Model Assessment of Insulin Resistance; LDL, Low-Density Lipoprotein.

Table S2. Comparison of grouped obesity classes by physical exam, laboratory, and imaging data of the entire study population.

	n	No Obesity ("0")	Class 1-3	Class 4-5	P value overall	P value 0 vs 1-3	P value 0 vs 4-5	P value 1-3 vs 4-5
		n=34,446	*n=19,539*	*n=808*				
**Body Mass Index [kg/m^2]**	54,793	25.0 [22.4;27.3]	34.3 [31.9;38.2]	54.2 [51.7;58.2]	<0.001	<0.001	<0.001	<0.001
**Waist Circumference [cm]**	53,031	88.2 [80.3;95.5]	111.0 [104.0;120.0]	146.0 [139.0;154.0]	<0.001	<0.001	<0.001	<0.001
**Truncal Fat Percentage [%]**	27,482	28.4 [22.5;34.4]	40.0 [34.5;44.6]	48.9 [45.2;52.2]	<0.001	<0.001	<0.001	<0.001
**Total Fat Percentage [%]**	26,802	29.2 [23.9;36.0]	40.1 [33.0;45.0]	48.8 [43.8;52.1]	<0.001	<0.001	<0.001	<0.001
**Lumbar Spine Bone Mineral Density [g/cm$^{2]}$**	27,492	1.0 [0.9;1.1]	1.0 [0.9;1.2]	1.2 [1.0;1.3]	<0.001	<0.001	<0.001	<0.001
**MASLD**	14,568				<0.001	<0.001	<0.001	<0.001
**Absent**		7,528 (88.3%)	3,006 (52.1%)	68 (24.5%)				
**Present**		997 (11.7%)	2,759 (47.9%)	210 (75.5%)				
**Hepatic Steatosis Grade 1+**	14,585				<0.001	<0.001	<0.001	<0.001
**Absent**		7,528 (88.1%)	3,006 (52.1%)	68 (24.5%)				
**Present**		1,014 (11.9%)	2,759 (47.9%)	210 (75.5%)				
**Hepatic Steatosis Grade 2+**	14,585				<0.001	<0.001	<0.001	<0.001
**Absent**		8,123 (95.1%)	4,034 (70.0%)	118 (42.4%)				
**Present**		419 (4.91%)	1,731 (30.0%)	160 (57.6%)				
**Hepatic Steatosis Grade 3**	14,585				<0.001	<0.001	<0.001	<0.001
**Absent**		8,205 (96.1%)	4,236 (73.5%)	122 (43.9%)				
**Present**		337 (3.9%)	1,529 (26.5%)	156 (56.1%)				
**Hepatic Fibrosis Stage 2+**	14,588				<0.001	<0.001	<0.001	<0.001
**Absent**		8,165 (95.6%)	4,803 (83.3%)	112 (40.1%)				
**Present**		377 (4.4%)	964 (16.7%)	167 (59.9%)				
**Hepatic Fibrosis Stage 3+**	14,588				<0.001	<0.001	<0.001	<0.001
**Absent**		8,337 (97.6%)	5,142 (89.2%)	138 (49.5%)				
**Present**		205 (2.4%)	625 (10.8%)	141 (50.5%)				

**Hepatic Fibrosis Stage 4**	14,588				<0.001	<0.001	<0.001	<0.001
**Absent**		8,451 (98.9%)	5,481 (95.0%)	189 (67.7%)				
**Present**		91 (1.1%)	286 (5.0%)	90 (32.3%)				
**Median Controlled Attenuation Parameter (CAP) [dB/m]**	14,585	230.0 [198.0;270.0]	298.0 [260.0;339.0]	348.0 [303.0;386.0]	<0.001	<0.001	<0.001	<0.001
**Median Liver Stiffness [kPa]**	14,588	4.6 [3.8;5.6]	5.50 [4.4;7.0]	10.0 [6.6;18.3]	<0.001	<0.001	<0.001	<0.001
**Metabolic Syndrome**	22,829				<0.001	<0.001	<0.001	<0.001
**Absent**		8,610 (61.9%)	2,276 (26.3%)	38 (13.7%)				
**Present**		5,299 (38.1%)	6,367 (73.7%)	239 (86.3%)				
**Prediabetes**	52,033				<0.001	<0.001	<0.001	<0.001
**Absent**		27,355 (83.8%)	13,194 (70.8%)	455 (60.2%)				
**Present**		5,284 (16.2%)	5,444 (29.2%)	301 (39.8%)				
**Diabetes**	52,193				<0.001	<0.001	<0.001	<0.001
**Absent**		30,540 (93.4%)	15,205 (81.2%)	517 (67.8%)				
**Present**		2,168 (6.6%)	3,517 (18.8%)	246 (32.2%)				
**Hemoglobin A1c [%]**	51,999	5.3 [5.1;5.6]	5.5 [5.3;6.0]	5.8 [5.4;6.4]	<0.001	<0.001	<0.001	<0.001
**Fasting Glucose [mg/dL]**	25,514	96.0 [89.6;103.0]	102.0 [94.6;113.0]	110.0 [98.0;131.0]	<0.001	<0.001	<0.001	<0.001
**Fasting Insulin [µU/mL]**	24,987	7.6 [5.1;11.4]	15.7 [10.3;23.6]	27.4 [17.6;39.3]	<0.001	<0.001	<0.001	<0.001
**HOMA-IR**	24,950	1.8 [1.2;2.8]	4.10 [2.6;6.6]	7.46 [4.76;12.0]	<0.001	<0.001	<0.001	<0.001
**Insulin Resistance**	24,950				<0.001	<0.001	<0.001	<0.001
**Absent**		10,888 (68.8%)	2,056 (23.4%)	9 (2.7%)				
**Present**		4,933 (31.2%)	6,743 (76.6%)	321 (97.3%)				
**Severe Insulin Resistance**	24,987				<0.001	<0.001	<0.001	<0.001
**Absent**		15,738 (99.3%)	8,404 (95.3%)	283 (85.5%)				
**Present**		103 (0.7%)	411 (4.7%)	48 (14.5%)				
**Fasting Triglycerides [mg/dL]**	22,823	91.0 [63.0;136.0]	119.0 [81.0;176.0]	103.0 [74.0;145.0]	<0.001	<0.001	<0.001	<0.001
**Fasting Total Cholesterol [mg/dL]**	51,258	187.0 [162.0;216.0]	192.0 [167.0;221.0]	177.0 [154.0;204.0]	<0.001	<0.001	<0.001	<0.001

**Fasting LDL Cholesterol [mg/dL]**	22,097	109.0 [87.0;133.0]	115.0 [94.0;138.0]	108.0 [87.0;125.0]	<0.001	<0.001	0.190	<0.001
**Fasting HDL Cholesterol [mg/dL]**	51,258	53.0 [44.0;65.0]	46.0 [39.0;55.0]	45.0 [39.0;55.0]	<0.001	<0.001	<0.001	0.460
**C-Reactive Protein (CRP) [mg/L]**	42,722	0.3 [0.1;1.0]	1.1 [0.4;3.5]	4.7 [1.5;11.3]	<0.001	<0.001	<0.001	<0.001
**Pulse [beats per minute]**	50,986	70.3 [64.0;80.0]	74.0 [66.0;82.0]	80.0 [70.0;88.0]	<0.001	<0.001	<0.001	<0.001
**Systolic Blood Pressure [mm Hg]**	46,156	115.0 [107.0;126.0]	121.0 [112.0;132.0]	123.0 [113.0;132.0]	<0.001	<0.001	<0.001	0.044
**Diastolic Blood Pressure [mm Hg]**	46,105	70.3 [63.3;77.3]	74.3 [67.3;82.0]	76.0 [68.7;84.0]	<0.001	<0.001	<0.001	<0.001

Adjusted p values are shown after pairwise Wilcoxon-Whitney-Mann rank-sum tests with Holm correction for multiplicity, where “0” (zero) equals the no obesity group. P value < 0.05 indicates statistical significance. Values are presented as median and in brackets the first and third quartiles. The number of subjects for which data were available is indicated in the second column. CAP, Controlled Attenuation Parameter; CRP, C-Reactive Protein; HDL, High-Density Lipoprotein; HOMA-IR, Homeostatic Model Assessment of Insulin Resistance; LDL, Low-Density Lipoprotein.

Table S3. Odds ratio of metabolic comorbidities per grouped obesity classes.

	n	No Obesity	Class 1-3	Class 4-5
		n=34,446	***n=19,539***	***n=808***
**MASLD**	14,568	Reference	6.9 [6.4;7.5]	23.3 [17.6;30.9]
**Hepatic Steatosis Grade 1+**	14,585	Reference	6.8 [6.3;7.4]	22.9 [17.3;30.4]
**Hepatic Steatosis Grade 2+**	14,585	Reference	8.3 [7.4;9.3]	26.3 [20.3;34.0]
**Hepatic Steatosis Grade 3**	14,585	Reference	8.8 [7.8;9.9]	31.1 [24.0;40.4]
**Hepatic Fibrosis Stage 2+**	14,588	Reference	4.4 [3.8;4.9]	32.3 [24.9;41.9]
**Hepatic Fibrosis Stage 3+**	14,588	Reference	4.9 [4.2;5.8]	41.6 [31.6;54.6]
**Hepatic Fibrosis Stage 4**	14,588	Reference	4.9 [3.8;6.2]	44.2 [32.0;61.2]
**Metabolic Syndrome**	22,829	Reference	4.5 [4.3;4.8]	10.2 [7.2;14.4]
**Prediabetes**	52,033	Reference	2.1 [2.1;2.2]	3.4 [3.0;4.0]
**Diabetes**	52,193	Reference	3.3 [3.1;3.5]	6.7 [5.7;7.9]
**Insulin Resistance**	24,950	Reference	7.2 [6.8;7.7]	78.7 [40.6;153.0]
**Severe Insulin Resistance**	24,987	Reference	7.5 [6.0;9.3]	25.9 [18.0;37.2]

Odds ratios and in brackets 95% confidence intervals are shown with the group without obesity serving as the reference group. MASLD, Metabolic Dysfunction-Associated Steatotic Liver Disease.

Table S4. Odds ratio of metabolic comorbidities per obesity classes.

	n	No Obesity	Class 1	Class 2	Class 3	Class 4	Class 5
		n=34,446	*n=10,820*	*n=5,231*	*n=3,488*	*n=650*	*n=158*
**MASLD**	14,568	Reference	4.7 [4.3;5.2]	8.5 [7.5;9.5]	14.1 [12.3;16.2]	30.2 [21.6;42.3]	10.7 [6.3;18.1]
**Hepatic Steatosis Grade 1+**	14,585	Reference	4.6 [4.2;5.1]	8.3 [7.4;9.4]	13.9 [12.1;15.9]	29.7 [21.2;41.6]	10.5 [6.2;17.8]
**Hepatic Steatosis Grade 2+**	14,585	Reference	5.1 [4.5;5.9]	10.4 [9.0;12.0]	16.8 [14.4;19.5]	30.8 [23.1;41.1]	14.7 [8.7;24.9]
**Hepatic Steatosis Grade 3**	14,585	Reference	5.3 [4.6;6.1]	10.7 [9.2;12.5]	18.6 [15.9;21.8]	35.8 [26.8;47.9]	18.4 [10.9;31.4]
**Hepatic Fibrosis Stage 2+**	14,588	Reference	2.2 [1.9;2.6]	5.1 [4.3;6.0]	10.3 [8.8;12.1]	28.7 [21.6;38.2]	52.2 [29.4;92.8]
**Hepatic Fibrosis Stage 3+**	14,588	Reference	2.5 [2.0;3.1]	5.0 [4.0;6.1]	12.6 [10.4;15.2]	35.5 [26.3;47.8]	77.2 [44.2;135.0]
**Hepatic Fibrosis Stage 4**	14,588	Reference	2.7 [2.0;3.6]	4.2 [3.1;5.8]	11.9 [9.0;15.6]	33.1 [23.0;47.6]	114.0 [65.5;200.0]
**Metabolic Syndrome**	22,829	Reference	3.8 [3.6;4.1]	5.2 [4.7;5.7]	6.9 [6.1;7.9]	9.8 [6.8;14.0]	14.2 [5.1;40.0]
**Prediabetes**	52,033	Reference	1.9 [1.8;2.0]	2.3 [2.2;2.5]	2.7 [2.5;2.9]	3.2 [2.7;3.7]	4.7 [3.4;6.5]
**Diabetes**	52,193	Reference	2.6 [2.4;2.8]	3.7 [3.4;4.0]	5.0 [4.6;5.5]	6.8 [5.7;8.1]	6.3 [4.4;8.8]
**Insulin Resistance**	24,950	Reference	5.2 [4.9;5.6]	9.7 [8.7;10.8]	16.9 [14.5;19.8]	78.5 [37.1;166.0]	79.5 [19.5;323.0]
**Severe Insulin Resistance**	24,987	Reference	5.1 [3.9;6.5]	8.1 [6.2;10.6]	14.2 [10.9;18.4]	27.3 [18.5;40.5]	21.2 [10.3;43.6]

Odds ratios and in brackets 95% confidence intervals are shown with the group without obesity serving as the reference group. MASLD, Metabolic Dysfunction-Associated Steatotic Liver Disease.

Supplementary Figures

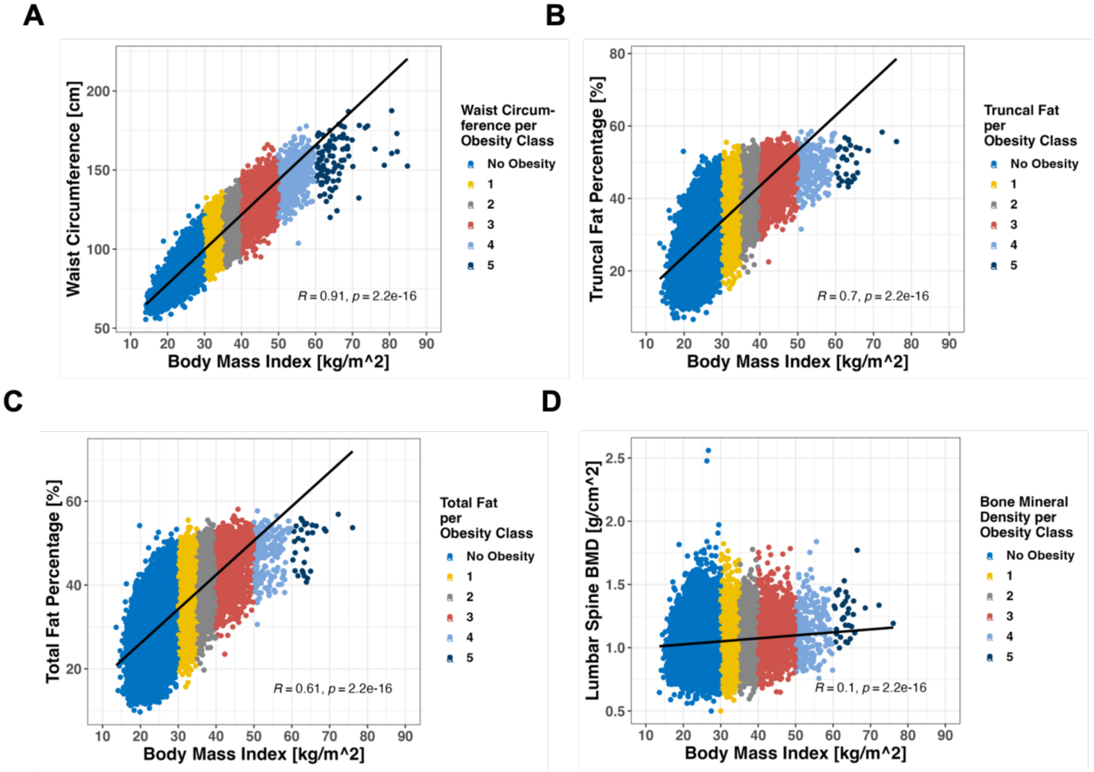

Figure S1. Waist circumference, truncal fat, total fat percentage, and lumbar spine bone mineral density correlate with body mass index. (A) Waist circumference per obesity class and body mass index (n=53,031). (B) Truncal fat percentage per obesity class and body mass index (n=27,482). (C) Total fat percentage per obesity class and body mass index (n=26,802). (D) Lumbar spine bone mineral density per obesity class and body mass index (n=27,492). The Pearson correlation coefficient *R* indicates strength of correlation. Statistical significance is indicated by $p<0.05$. BMD, bone mineral density.

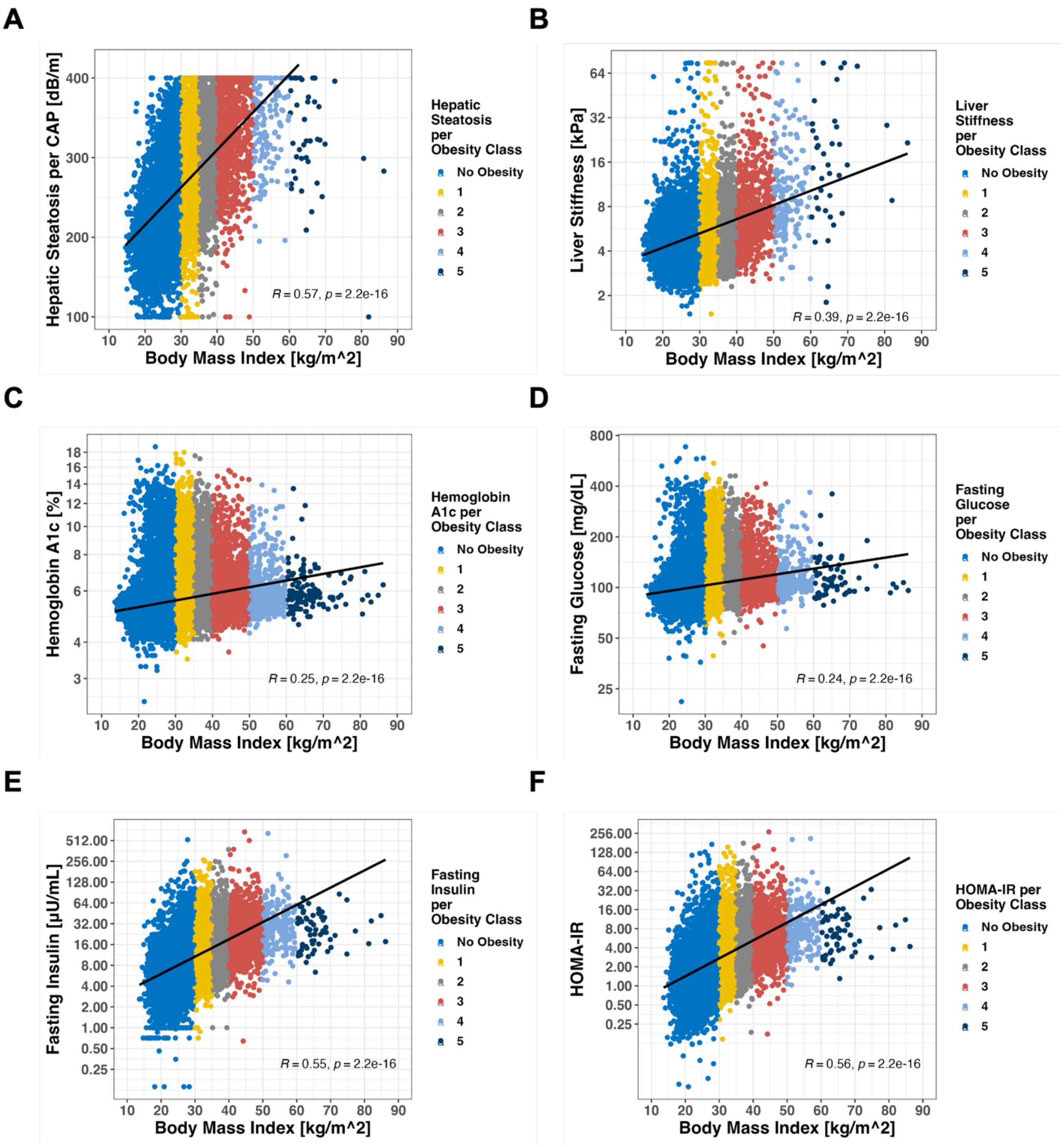

Figure S2. Markers of liver disease, diabetes mellitus, and insulin resistance correlate body mass index. (A) Hepatic steatosis per CAP per obesity class and body mass index (n=14,585). (B) Liver stiffness per obesity class and body mass index (n=14,588). (C) Hemoglobin A1c per obesity class and body mass index (n=51,999). (D) Fasting glucose per obesity class and body mass index (n=25,514). (E) Fasting insulin

per obesity class and body mass index (n=24,987). (F) HOMA-IR per obesity class and body mass index (n=24,950). The Pearson correlation coefficient R indicates strength of correlation. Statistical significance is indicated by $p<0.05$. CAP, Controlled Attenuation Parameter; HOMA-IR, Homeostatic Model Assessment of Insulin Resistance.

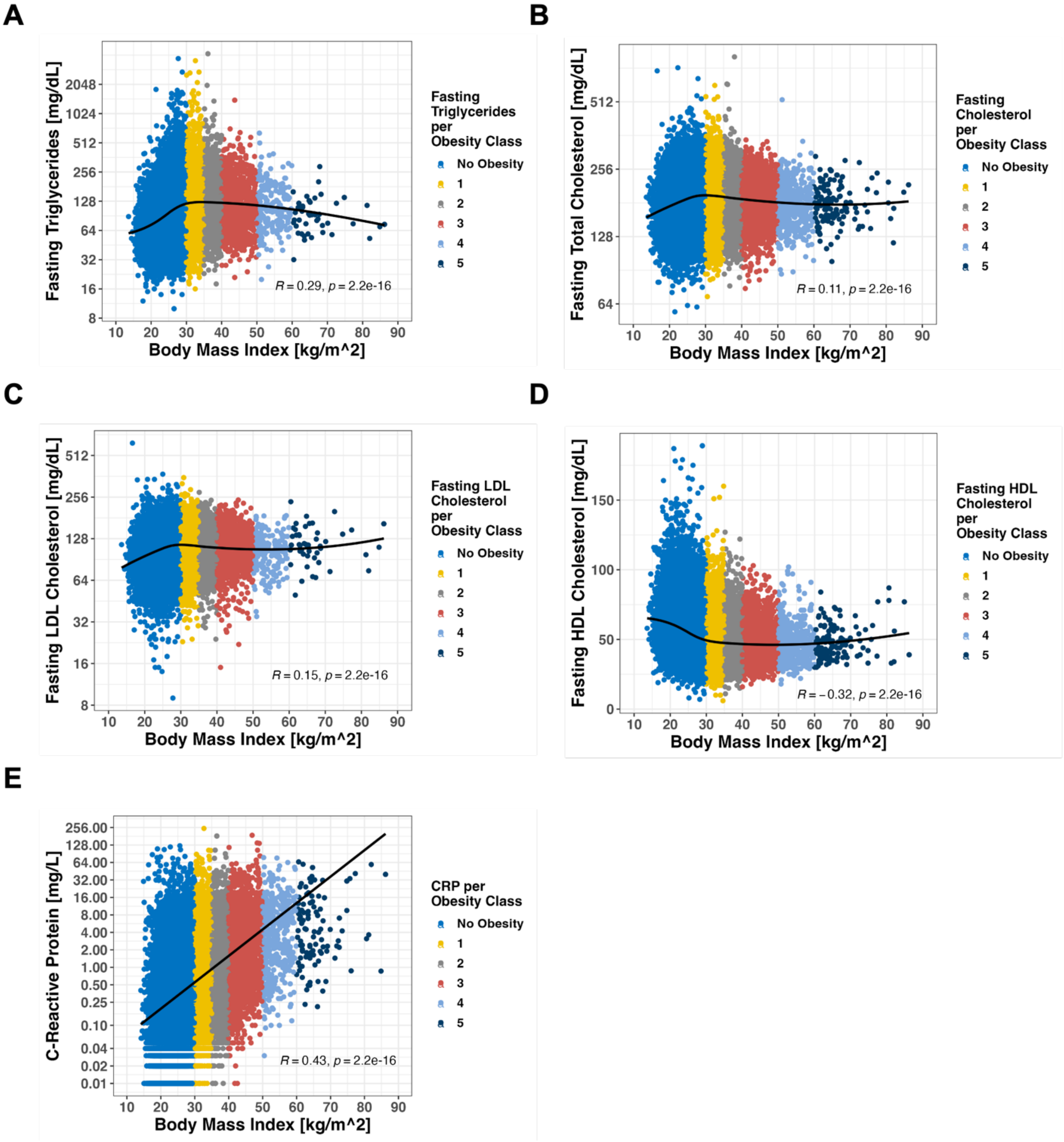

Figure S3. Markers of dyslipidemia tend to be most severe in class 1 obesity. (A) Fasting triglycerides per obesity class and body mass index (n=22,823). (B) Fasting total cholesterol per obesity class and body mass index (n=51,258). (C) Fasting LDL cholesterol per obesity class and body mass index (n=22,097). (D) Fasting HDL cholesterol per obesity class and body mass index (n=51,258). (E) C-Reactive Protein

per obesity class and body mass index (n=42,722). The Spearman correlation coefficient *R* indicates strength of correlation. Statistical significance is indicated by $p<0.05$. HDL, High-Density Lipoprotein; LDL, Low-Density Lipoprotein.

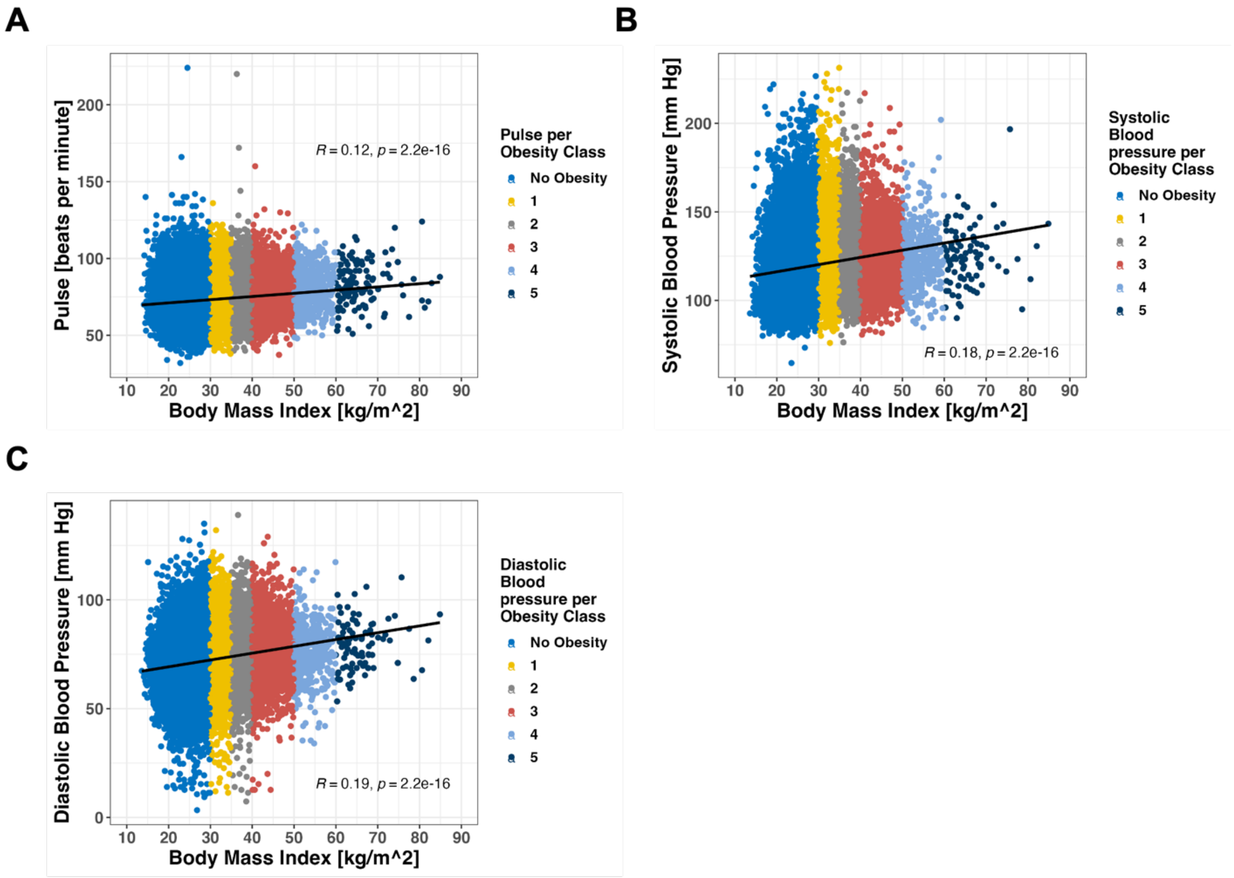

Figure S4. Pulse and blood pressure correlate body mass index. (A) Pulse per obesity class and body mass index (n=50,986). (B) Systolic blood pressure per obesity class and body mass index (n=46,156). (C) Diastolic blood pressure per obesity class and body mass index (n=46,105). The Pearson correlation coefficient *R* indicates strength of correlation. Statistical significance is indicated by $p<0.05$.
